# The Two-Component Sensor Kinase TcsC and Its Role in Stress Resistance of the Human-Pathogenic Mold *Aspergillus fumigatus*

**DOI:** 10.1371/journal.pone.0038262

**Published:** 2012-06-04

**Authors:** Allison McCormick, Ilse D. Jacobsen, Marzena Broniszewska, Julia Beck, Jürgen Heesemann, Frank Ebel

**Affiliations:** 1 Max-von-Pettenkofer-Institut, Ludwig-Maximilians-University, Munich, Germany; 2 Department for Microbial Pathogenicity Mechanisms, Leibniz Institute for Natural Product Research and Infection Biology, Jena, Germany; 3 Center of Integrated Protein Science (Munich) at the Faculty of Medicine of the Ludwig-Maximilians-University, Munich, Germany; Montana State University, United States of America

## Abstract

Two-component signaling systems are widespread in bacteria, but also found in fungi. In this study, we have characterized TcsC, the only Group III two-component sensor kinase of *Aspergillus fumigatus*. TcsC is required for growth under hyperosmotic stress, but dispensable for normal growth, sporulation and conidial viability. A characteristic feature of the Δ*tcsC* mutant is its resistance to certain fungicides, like fludioxonil. Both hyperosmotic stress and treatment with fludioxonil result in a TcsC-dependent phosphorylation of SakA, the final MAP kinase in the high osmolarity glycerol (HOG) pathway, confirming a role for TcsC in this signaling pathway. In wild type cells fludioxonil induces a TcsC-dependent swelling and a complete, but reversible block of growth and cytokinesis. Several types of stress, such as hypoxia, exposure to farnesol or elevated concentrations of certain divalent cations, trigger a differentiation in *A. fumigatus* toward a “fluffy” growth phenotype resulting in white, dome-shaped colonies. The Δ*tcsC* mutant is clearly more susceptible to these morphogenetic changes suggesting that TcsC normally antagonizes this process. Although TcsC plays a role in the adaptation of *A. fumigatus* to hypoxia, it seems to be dispensable for virulence.

## Introduction

*Aspergillus fumigatus* is a mold causing severe and systemic infections in immunocompromised patients [1]. The high mortality of these infections is largely due to the limited therapeutic options. Since *A. fumigatus* seems to lack sophisticated virulence factors, alternative therapeutic targets must be considered. The ability to respond to a plethora of environmental changes and to cope with different stress situations is vital for growth and survival of all microorganisms. This applies in particular to microbial pathogens that have to adapt to changing environments and a hostile immune response during colonization and invasion of the host. In fungi, sensing and responding to environmental stress is mediated by a set of receptors that are linked to a network of down-stream signaling pathways [2]. Interference with these signal transduction cascades can impede the fungal adaptation to stress and is considered a promising option to identify novel therapeutic targets. However, this approach is hampered by the conservation of many central signaling molecules in fungi and humans.

In bacteria sensing and processing of stress signals relies largely on two-component systems (TCS) that consist of a sensor histidine kinase and a response regulator. In fungi and other eukaryotes, hybrid histidine kinases (HHK) integrate both functions in a single protein. Fungal TCS are multistep phospho-relays composed of a sensor kinase (HHK), a histidine-containing phosphotransfer protein (HPt) and one or two response regulators. HHK are conserved within the fungal kingdom and depending on the species they govern the response to various stress signals, including osmotic stress, oxidative stress, hypoxia, resistance to anti-fungals and sexual development [3], [4]. In contrast to other signaling molecules, TCS are attractive candidates for new therapeutic targets since they contribute to the virulence of fungal pathogens and are not found in vertebrates [3], [5].

In fungi, eleven families of HHK have been described according to their protein sequence and domain organization [6]. Of several potential HHK present in the genome of *A. fumigatus* only two have been studied so far. Deletion of the Group VI HHK gene *tcsB* (AFUA_2G00660) had no severe impact on growth and stress resistance of *A. fumigatus*, but led to a slightly increased sensitivity to SDS [7]. A mutant in the Group IV HHK *tcsA*/*fos1* (AFU6G10240) showed normal growth, no increased sensitivity to osmotic stress, but resistance to dicarboximide fungicides, like iprodione, and enzymatic cell wall degradation [8]. This is remarkable, since dicarboximide fungicides commonly target Group III HHK [9]. Several lines of evidence link Group III HHK to the high osmolarity glycerol (HOG) pathway that was initially described as a signaling module enabling yeasts to adapt to high external osmotic pressure [10]. However, recent evidence suggests that in pathogenic fungi the HOG pathway is furthermore involved in the response to diverse kinds of stress [4].

In this study, we have analyzed TcsC, the sole representative of the Group III HHK in *A. fumigatus*. Group III HHK are found in bacteria, plants and fungi. They contain a characteristic cluster of HAMP domains that mediate signaling in histidine kinases, adenylyl cyclases, methyl-accepting chemotaxis proteins and certain phosphatases. Conformational changes in the spatial organization of the amphipathic helices in HAMP domains allow two conformations that either activate or inactivate the kinase activity of the output domain [11]. Single HAMP domains of membrane-bound HHK are found in close proximity to the membrane-spanning segment and transduce signals from the external input to the internal output domain. Group III HHK contain clusters of 4-6 HAMP domains, that according to a model developed recently for the osmo-tolerant yeast *Debaryomyces hansenii,* form a functional unit that is able to sense external signals. Changes in external osmolarity are supposed to alter the pattern of HAMP domain interactions and thereby modulate the inherent kinase activity of the protein [12]. The facts that Group III HHK are exclusively found in fungi and that certain fungicides can activate these sensor kinases in an uncontrolled and harmful manner makes them a potential Achilles heel of fungal pathogens that merits further investigations.

## Results

### The Group III HHK TcsC of *A. fumigatus*

The genome of *A. fumigatus* contains only one putative Group III HHK (AFU2G03560). The corresponding protein comprises a histidine kinase acceptor domain, a histidine kinase-like ATPase domain, a receiver domain and six HAMP domains. It lacks a transmembrane segment and is presumably localized in the cytoplasm. We designated this protein **T**wo-**c**omponent **s**ystem protein **C** (**TcsC**) following the nomenclature of the previously studied *Aspergillus* TCS sensor kinases TcsA (Fos-1; AFU6G10240) and TcsB (AFU2G00660) [7], [8], [13].

### Generation and Characterization of a Δ*tcsC* Mutant

**Figure 1:**
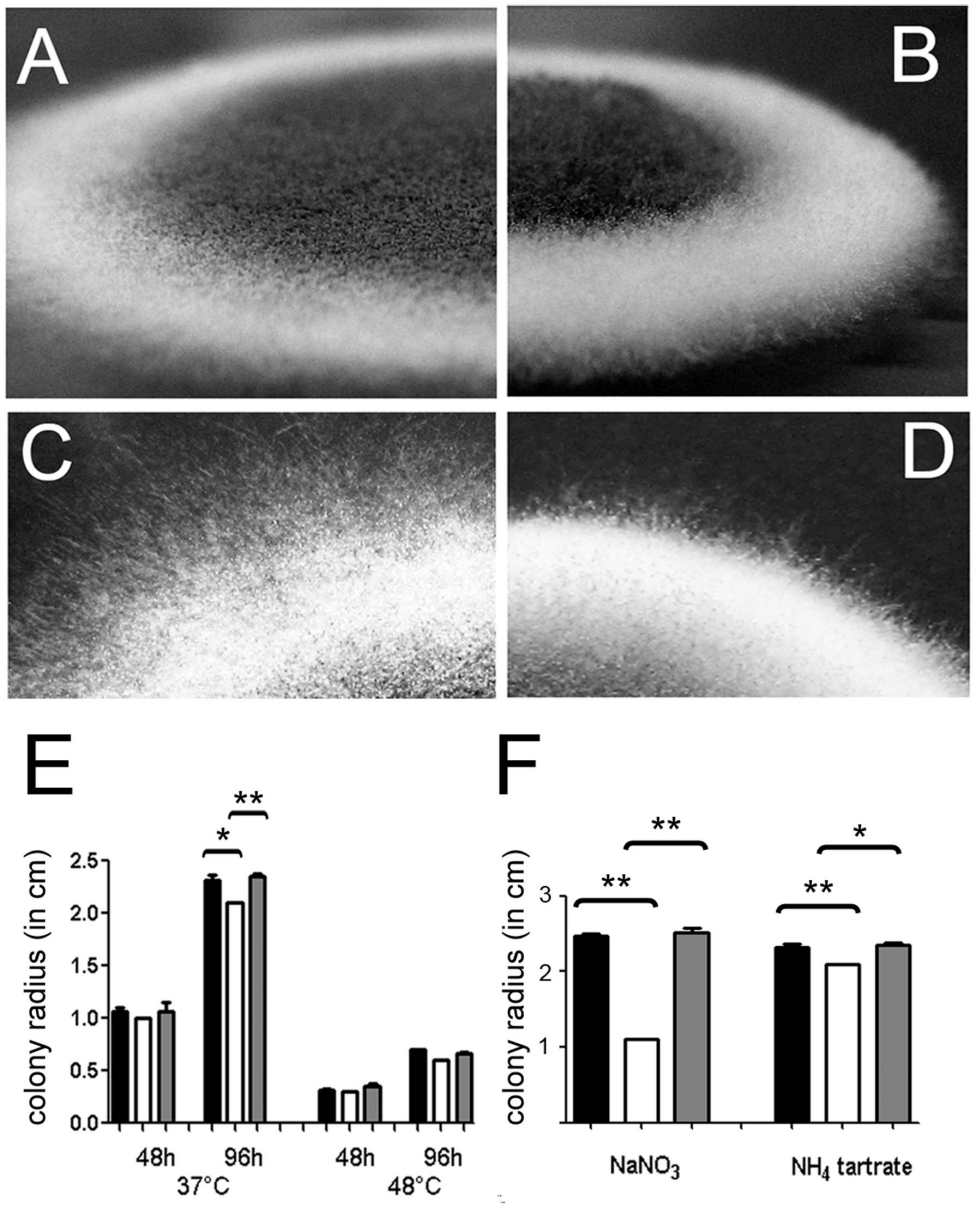
Growth of the Δ*tcsC* mutant. Colonies of the AfS35 wild type and the Δ*tcsC* mutant grown for 72 h on AMM plates are shown in panels A/C and B/D, respectively. Magnifications of the edge of the colonies are depicted in panels C and D. Note the reduced number of extending hyphae in the mutant. Panel E: Quantification of the radial growth of AfS35 (black), Δ*tcsC* mutant (white) and complemented mutant colonies (gray) on AMM plates after 48 h and 96 h at 37°C or 48°C. Panel F: Quantification of the radial growth after 96 h of AfS35 (black), Δ*tcsC* mutant (white) and complemented mutant colonies (gray) on AMM plates supplemented with 1.4 M NaNO3 or 0.2 M ammonium tartrate at 37°C. The experiments shown in panels E and F were done in triplicate. Standard deviations are indicated. Student’s *t*-test: *p<0.005; **p<0.001.

To analyze the function of TcsC, we deleted the gene and complemented the mutant by ectopic insertion of the *tcsC* gene under control of its native promoter. The complementation procedure and the analysis of the genotype of the resulting strain are shown in Figure S1. On AMM, YG or Sabouraud medium the mutant grew well, but the colonies had a distinct appearance characterized by a broader white rim and fewer extending hyhae at the periphery (Figure 1A to D), whereas the complemented strain was indistinguishable from the wild type (data not shown). At 48°C growth of the mutant was comparable to the controls (Figure 1E) demonstrating that it is not particularly sensitive to temperature stress. Radial growth of the Δ*tcsC* mutant was slightly slower on AMM supplemented with ammonium tartrate (Figure 1E), whereas a remarkable reduction in growth was found on AMM plates supplemented with NaNO_3_ instead of ammonium tartrate. This defect was not observed for the complemented strain indicating that TcsC is required for normal growth with nitrate as sole nitrogen source (Figure 1F).

In *A. nidulans* deletion of the homologous *nikA* gene had severe consequences for the production and viability of asexual spores [14], [15], [16]. We therefore compared sporulation and conidial viability of the Δ*tcsC* mutant and its parental strain. After four days at 37°C both strains produced a confluent and sporulating mycelial layer. No obvious difference in sporulation was apparent and this was confirmed by determining the conidial yield per cm^2^ (mutant: 9.7±0.8×10^7^, parental strain: 9.3±0.8×10^7^). Conidia of the Δ*nikA* gene lose their viability within a few days when stored in water at 4°C. In contrast, conidia of the Δ*tcsC* mutant remained fully viable after storage for one month (mutant: 93.7%±3.0%, parental strain: 95.5%±3.0%). Thus, deletion of the Group III HHK gene in *A. fumigatus* does not affect sporulation or conidial viability, thus disclosing a remarkable difference between the two homologous sensor kinases in *A. fumigatus* and *A. nidulans*.

**Figure 2:**
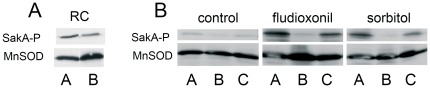
The role of TcsC in the phosphorylation of SakA. Protein extracts of resting conidia (RC)(panel A) and germlings (panel B) were analyzed by immunoblot using specific antibodies to phosphorylated SakA and as a loading control mitochondrial MnSOD. Extracts were prepared from germlings treated with 10 µg/ml fludioxonil and 1.2 M sorbitol for 2 and 20 min, respectively. A: parental strain AfS35, B: Δ*tcsC* mutant, C: complemented mutant.

Conidial viability in *A. nidulans* was recently shown to depend on the presence phosphorylated SakA in resting conidia [17]. Several Group III HHK have been linked to the HOG pathway and shown to influence the phosphorylation state of HOG proteins, like *Aspergillus* SakA. In immunoblot experiments we detected only a slight decrease in the level of SakA phosphorylation in resting conidia of the Δ*tcsC* mutant when compared to its parental strain (Figure 2A), demonstrating that TcsC is not essentially required for SakA phosporylation in resting conidia.

**Figure 3:**
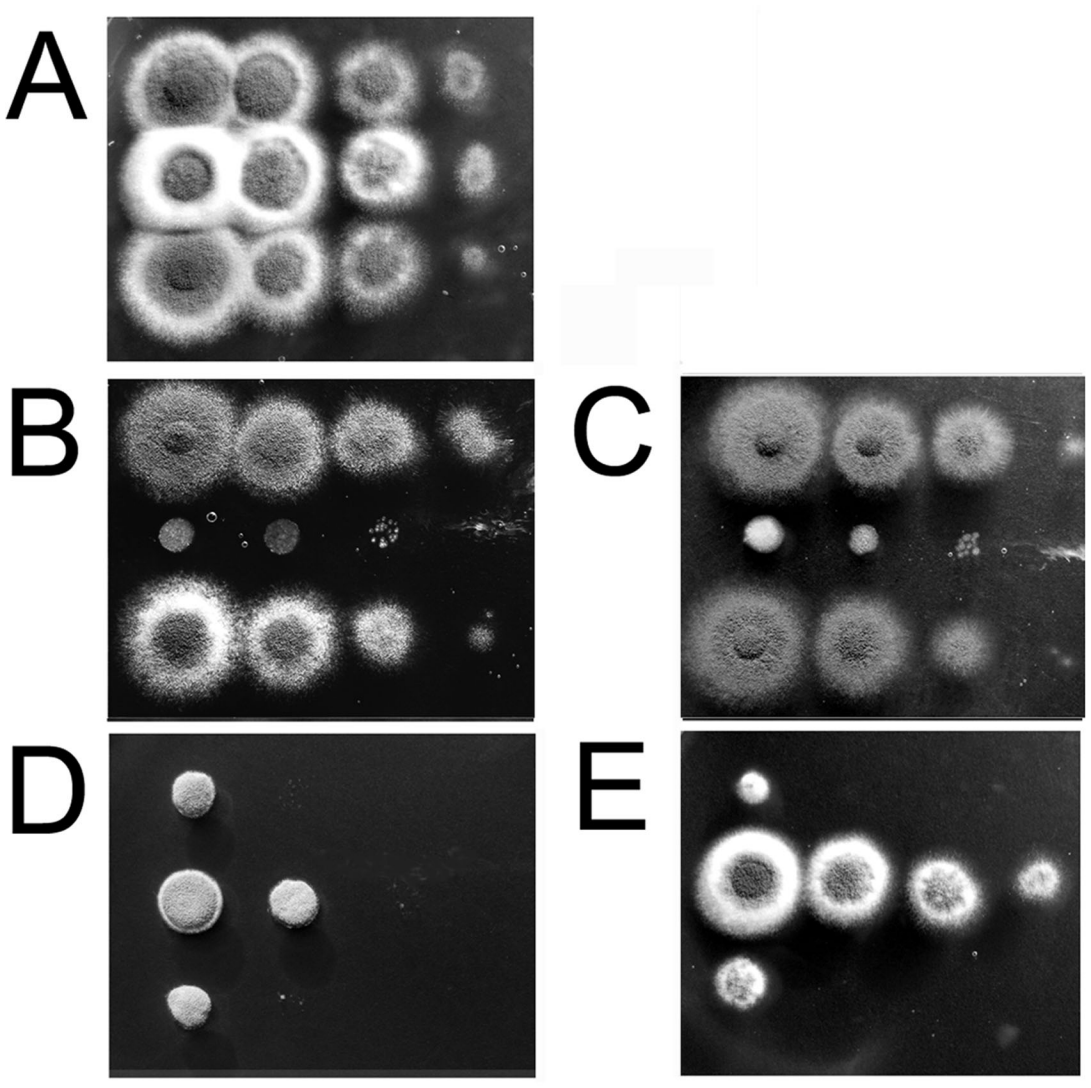
The Δ*tcsC* mutant is sensitive to hyperosmotic stress and resistant to fludioxonil. Drop dilution assays were performed on AMM plates (supplemented with ammonium). Panel A: control; B: 1.2 M sorbitol; C: 1 M KCl; D: 100 µg/ml congo red; E: 1 µg/ml fludioxonil. The depicted colonies were obtained after 48 h at 37°C. Top: AfS35; middle: Δ*tcsC*; bottom: complemented strain.

Group III HHK have been shown to be required for resistance to osmotic stress in several fungi, but not in *A. nidulans*. Our data revealed a strong growth inhibition of the Δ*tcsC* mutant under hyperosmotic stress, e.g. on plates containing 1.2 M sorbitol (Figure 3B), 1 M KCl (Figure 3C) and 1 M NaCl (data not shown). This demonstrates that TcsC is clearly important for adaptation to high osmolarity. Immunoblot analysis revealed that SakA phosphorylation is much weaker in germlings than in resting conidia (Figure 2A and B). However, both 1.2 M sorbitol and the antifungal agent fludioxonil induced SakA hyper-phosphorylation in a TcsC-dependent manner (Figure 2B). Thus, TcsC is required for activation of the HOG pathway by hyperosmotic stress and the phenylpyrrole antifungal agent fludioxonil.

We found no evidence for an enhanced sensitivity of the Δ*tcsC* mutant to calcofluor white, several clinically relevant antifungals (amphotericin B, posaconazol and caspofungin), pH (pH 5-9), temperature (20°C–48°C) or oxidative stress (H_2_O_2_ and t-BOOH) (data not shown). In fact, the mutant turned out to be slightly more resistant to the cell wall stressor congo red and UV light (Figure 3D and data not shown). Thus, TcsC activity is required for adaptation to hyper-osmotic stress, but is not essential for the general stress response.

### TcsC is Essential for the Fungicidal Acitivity of Fludioxonil and Related Compounds

An interesting feature of Group III HHK mutants is their resistance to fludioxonil and related fungicides. Accordingly, the Δ*tcsC* mutant grew normally in liquid medium containing 10 µg/ml fludioxonil, whereas growth of the wild type was completely abrogated at 1 µg/ml fludioxonil (data not shown). This phenotype was also evident in drop dilution assays on plates supplemented with fludioxonil (1 µg/ml; Figure 3E) or the functionally related fungicides quintozene (25 µg/ml) and iprodione (25 µg/ml) (Figure S2 B and C, respectively).

**Figure 4:**
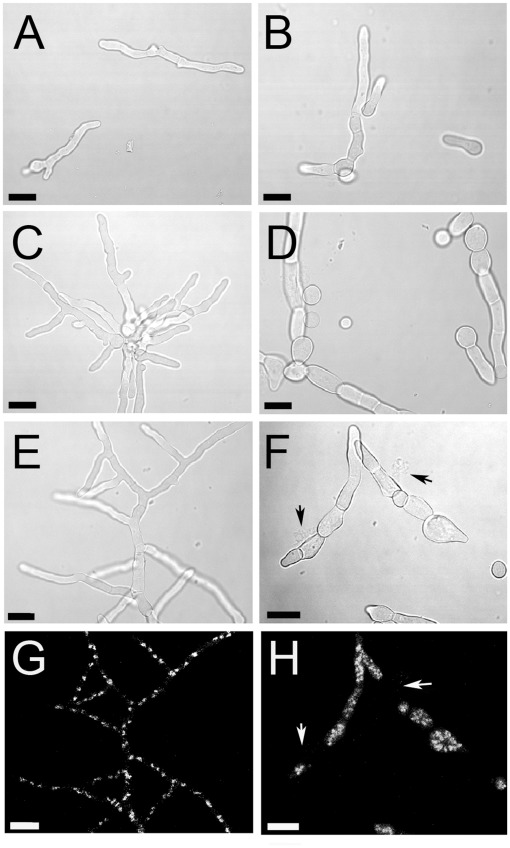
Impact of fludioxonil on *A. fumigatus* germ tubes. Conidia of the Δ*tcsC* mutant (panels A, C, E, G) and its parental strain AfS35 (panels B, D, F, H) were seeded on glass cover slips and incubated overnight in AMM at 30°C. The resulting germ tubes were treated with 1 µg/ml fludioxonil for 2 h (A, B), 4 h (C, D) and 6 h (E–H) at 37°C. A DAPI staining is shown in panels G and H. Arrows indicate lysed cells that lack intracellular nuclei and are associated with amorphous extracellular material. All bars represent 10 µm.

To obtain more information on the impact of fludioxonil at the level of individual cells, germlings were incubated in the presence of 1 µg/ml fludioxonil. No obvious morphological changes were apparent after 2 h (Figure 4A and B), but 4 h and 6 h after addition of fludioxonil growth of the wild type (Figure 4D and F) and the complemented mutant (data not shown) stopped and the cells began to swell, whereas the growth and morphology of the Δ*tcsC* mutant remained normal (Figure 4C and E). Similar results were obtained with 25 µg/ml iprodione (data not shown). DAPI staining of germlings treated with fludioxonil for 6 h revealed a normal distribution of nuclei in hyphae of the mutant (Figure 4G), but an unusually high number of nuclei in the swollen cells of the wild type (Figure 4H) and the complemented mutant (data not shown).

We also analyzed the impact of fludioxonil on the germination of resting conidia. Spores were incubated in medium supplemented with 1 µg/ml fludioxonil. After 28 h, the wild type produced only small germlings (Figure S3 A and B), while abundant hyphae were found in the fludioxonil-treated Δ*tcsC* mutant and an untreated wild type control (data not shown). Thus, germination of wild type spores was impaired, but not completely abolished by fludioxonil. An additional 18 h incubation in fludioxonil yielded cells whose growth was arrested and these exhibited irregular, swollen morphologies (Figure S3 C-F). As observed for germlings, fludioxonil treatment during germination resulted in unusually high numbers of nuclei that were often clustered in the cytoplasm (Figure S3 A, C and E). Only few fludioxonil-treated cells showed signs of leakage after 46 h (data not shown). We therefore replaced the medium and incubated the cells for another 15 h without fludioxonil to analyze their ability to recover. Although fludioxonil had induced severe morphological changes the cells were able to restore growth and the resulting hyphae had a normal appearance and a normal distribution and number of nuclei ( G and H).

### The Role of TcsC in the *fluffy* Growth Phenotype in *A. fumigatus*

**Figure 5:**
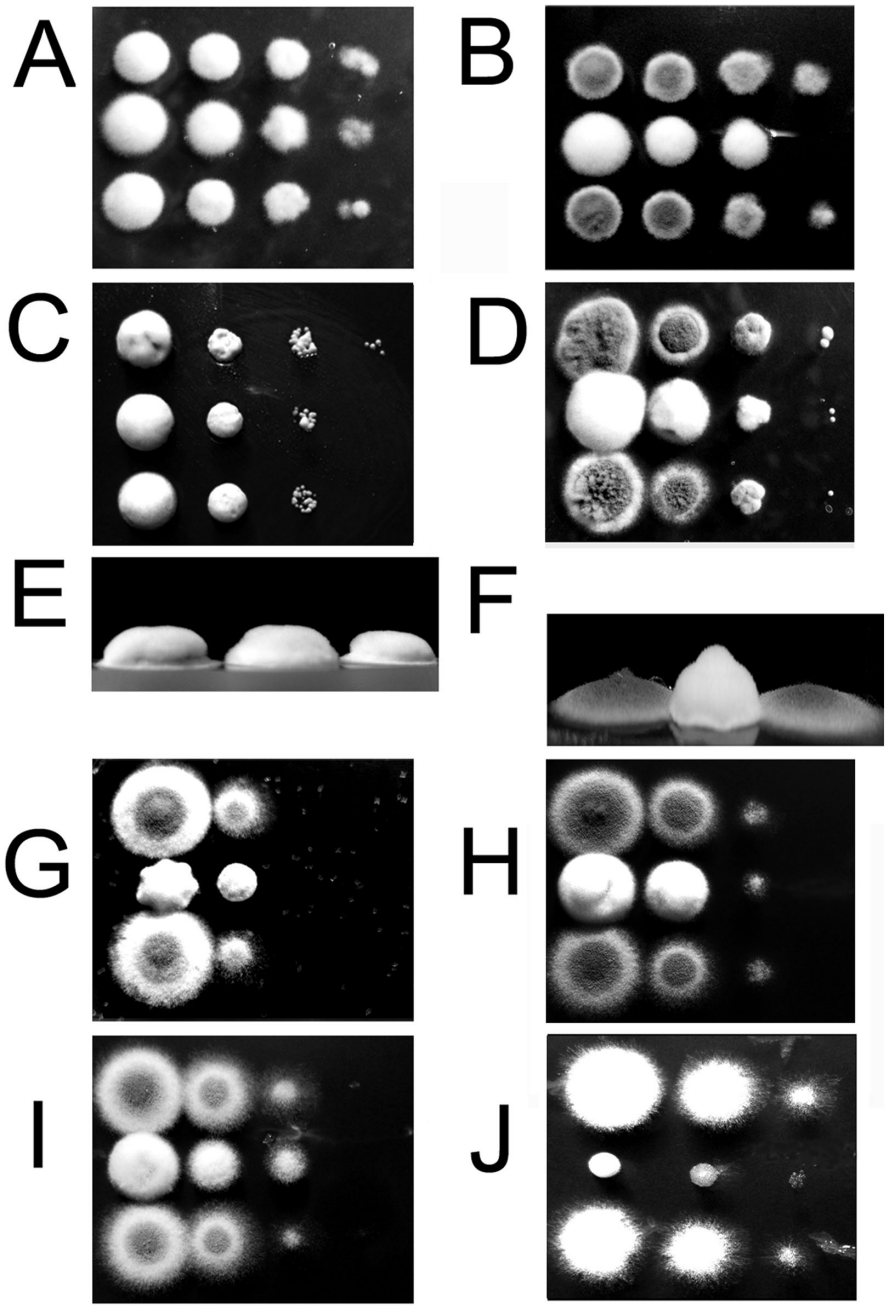
The role of TcsC in the stress-induced developmental program leading to a fluffy growth phenotype. Drop dilution assays were performed on AMM plates (supplemented with ammonium). Panel A: 1% oxygen; B: 2% oxygen; C: 2 mM farnesol; D: 200 µM farnesol; E: 2 mM farnesol; F: 100 mM MgSO_4_; G: 100 mM CaCl_2_; H: 100 mM MgSO_4_; I: 50 mM CaCl_2_; J: 500 mM CaCl_2_. Side views of colonies from C and D are shown in panels E and F. The depicted colonies were photographed after 48 h at 37°C. AfS35 (top/left); Δ*tcsC* (middle); complemented strain (bottom/right).

Tco1, the Group III HHK of *Cryptococcus neoformans*, is required for growth under hypoxic conditions [18]. Oxygen limitation is also encountered by *A. fumigatus* during infection and it was recently shown that its ability to grow under hypoxic conditions is a prerequisite for virulence [19]. Adaptation of *A. fumigatus* to 1% oxygen results in colonies that are characterized by a massive production of aerial hyphae, resulting in a dome-shaped morphology, and a complete lack of sporulation (Figure 5A). At 1% oxygen the Δ*tcsC* mutant was indistinguishable from the control strains with respect to growth and colony morphology. At 2% oxygen flat and sporulating colonies were found for the control strains, whereas the mutant colonies remained white and dome-shaped (Fig. 5B). Similar *A. nidulans* colonies, also characterized by the formation of abundant aerial hyphae and the lack of sporulation, were described previously as having a ‘fluffy’ developmental phenotype [20]. Thus, oxygen limitation seems to activate a specific morphogenetic program and the threshold level of hypoxic stress required to trigger this developmental process is clearly lower in the Δ*tcsC* mutant.

A fluffy phenotype is also apparent in the presence of 2 mM of the acyclic sesquiterpene alcohol farnesol (Figure 5C and E; [21]). Titration of farnesol revealed that at lower concentrations the fluffy growth was restricted to the Δ*tcsC* mutant (Figure 5D). Thus, the absence of TcsC renders *A. fumigatus* more sensitive to oxygen limitation and farnesol. Further experiments revealed a third trigger for fluffy growth in *A. fumigatus*. White, dome-shaped colonies of the mutant, but not of the control strains were obtained on plates containing 100 mM CaCl_2_ and 100 mM MgCl_2_ (Figure 5G and data not shown). This phenotypic switch was also induced by 100 mM MgSO_4_ (Figure 5F and H), but not by 200 mM NaCl (data not shown), indicating that divalent cationic ions, but not the slight increase in osmolarity or elevated chloride concentration induced the fluffy growth. The phenotypic differentiation was already obvious with 50 mM CaCl_2_ (Figure 5I), and could be enforced by addition of 20 µM farnesol, which *per se* had no impact on the colony morphology (data not shown), suggesting a synergistic mode of action for these stimuli. A further increase of the calcium concentration to 500 mM induced the fluffy growth phenotype in the control strains, but concomitantly abrogated growth of the mutant (Figure 5J). Thus, oxygen limitation, farnesol and divalent cations activate the fluffy developmental program and the lack of *tcsC* renders cells more susceptible to this developmental reprogramming.

**Figure 6:**
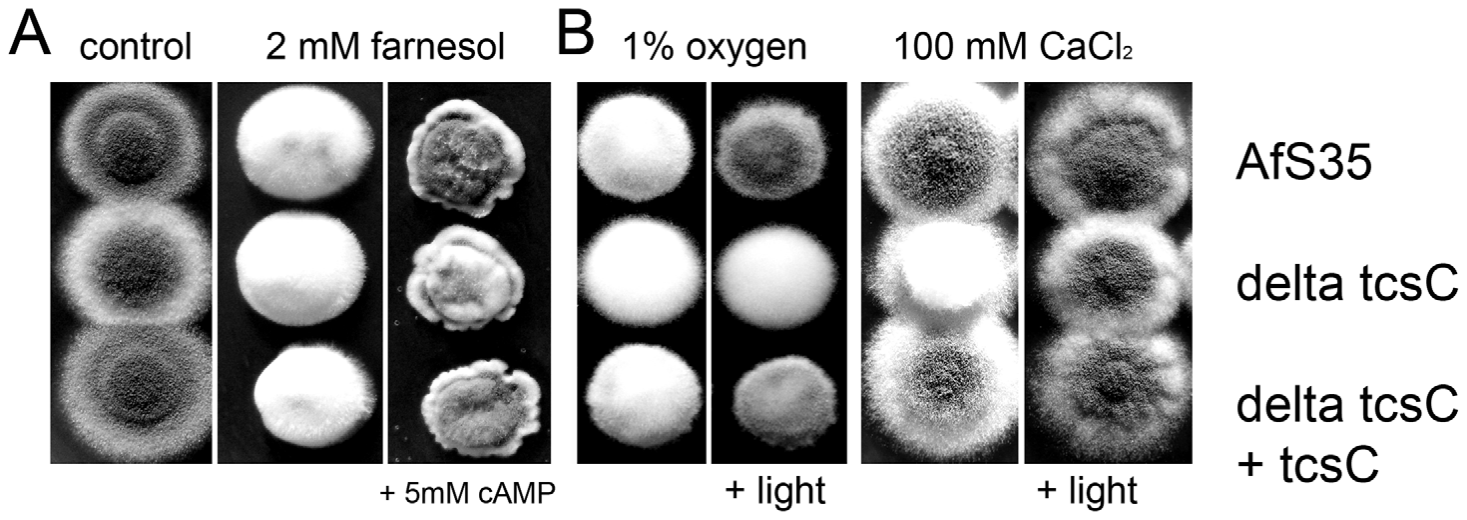
The impact of cAMP and light on the fluffy growth phenotype. Drop dilution assays were performed on AMM plates (supplemented with ammonium). The plates were supplemented or treated as indicated and incubated in incubator. When indicated plates were incubated under white light produced by an LED light source. Pictures were taken after 48 h at 37°C.

The fluffy growth phenotype in *A. nidulans* is regulated by a heterotrimeric G protein that has been functionally linked to the cAMP-dependent protein kinase pathway [22], [23]. For *A. fumigatus,* addition of 5 mM cAMP partially rescued the sporulation defect caused by farnesol (Figure 6A), but not that triggered by 100 mM CaCl_2_ or hypoxia (1% oxygen) (data not shown). We also tested the influence of light that stimulates sporulation in many fungi. Exposure of colonies to white light rescued the sporulation defect induced by 1% oxygen in the parental and the complemented strain, but not in the Δ*tcsC* mutant. Moreover, light also reduced the formation of aerial hyphae and resulted in colonies with a normal appearance (Figure 6B). 100 mM CaCl_2_ or 2% oxygen are weaker activators of the fluffy program. They only influence the growth of the Δ*tcsC* mutant and this effect can also be prevented by light (Figure 6B and data not shown). The impact of light on the farnesol-induced sporulation defect could not be analyzed due to the known sensitivity of this agent to light. Thus, light and cAMP can antagonize the development towards a fluffy growth phenotype. In doing so cAMP was only able to neutralize the effect of farnesol, whereas light seems to have a broader impact.

### Analysis of the Virulence of the Δ*tcsC* Mutant

**Figure 7:**
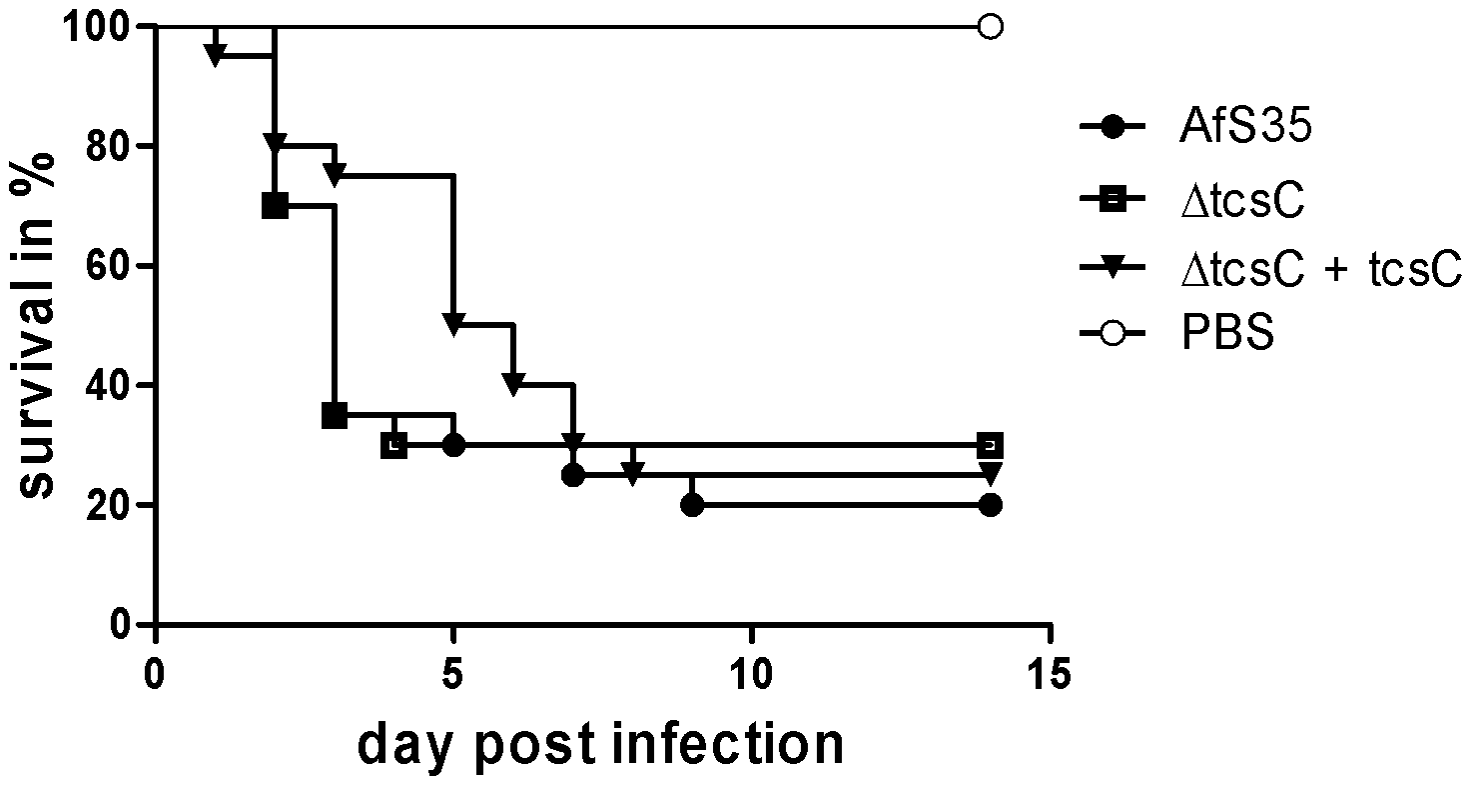
Infection of immuno-compromized mice. Intranasal infection of cortisone-acetate treated mice infected with 1×10^6^ conidia of the Δ*tcsC* mutant (n = 20), the parental strain AfS35 (n = 20) and the complemented strain (n = 20). Controls received PBS only. Survival of mice is shown over time.

The ability to respond to certain kinds of stress is clearly impaired in the Δ*tcsC* mutant. In order to investigate whether this negatively affects its virulence potential, cortisone-acetate treated mice were infected via the intra-nasal route. Survival of mice infected with the Δ*tcsC* mutant was comparable to those infected with the control strains (Figure 7) and the histological analysis of samples from the lungs of mice that succumbed to infection also revealed no apparent differences (data not shown). A normal virulence was furthermore observed in a alternative infection model using embryonated eggs [24](data not shown).

## Discussion

In an often hostile environment pathogenic microorganisms rely on the ability to sense and respond to environmental changes. Two-component signaling (TCS) systems are sensing entities that are abundant in bacteria, but also found in fungi and plants. Because they are absent in mammals, TCS systems and their hybrid histidine kinases (HHK) are potential targets for novel anti-microbial strategies. Group III HHK are predicted to localize in the cytoplasm, but are nevertheless supposed to sense changes in the environment. The resulting signals are then transferred via a phospho-relay system to two response regulators that directly or indirectly trigger an appropriate transcriptional response [4]. In this study we have analyzed TcsC, the only Group III HHK of the pathogenic mold *A. fumigatus*. Deletion of the homologous *nikA* gene in *A. nidulans* has been reported to cause a significantly reduced growth on solid medium [14], [15], whereas the Δ*tcsC* mutant grows normally on complex media and on minimal medium (AMM) supplemented with ammonium. Growth was however impaired on AMM supplemented with nitrate, suggesting that TcsC is required for efficient nitrogen assimilation. In this context it is noteworthy that the growth defect of the Δ*nikA* mutant was observed using minimal medium with nitrate as the sole nitrogen source [15] and it would be interesting to test the growth of this mutant on a medium containing ammonium.

Although Group III HHK are often linked to the high osmolarity glycerol (HOG) pathway, their relevance for adaptation to hyperosmotic stress seems to vary in different fungi. While the Δ*nikA* mutant showed a normal ability to adapt to hyperosmotic stress [15], the Δ*tcsC* mutant turned out to be highly sensitive. Another striking difference between both mutants exists with respect to their conidial viability. Conidia of the Δ*nikA* mutant showed a dramatic loss of viability when stored in water for several days [14], [15], whereas conidia of the Δ*tcsC* mutant remained fully viable upon storage for several weeks. Thus, TcsC and NikA although closely related, appear to differ in their biological activities.

A characteristic feature of mutants lacking Group III HHK is their resistance to fungicides, like fludioxonil. These compounds are currently used in agriculture, but are also of potential interest for the development of novel therapeutic anti-fungals. Their mode of action is unique in that they activate a fungal signaling process, the HOG pathway. The hallmark of this activation is the phosphorylation and subsequent translocation of SakA/Hog1 to the nucleus [10]. In *A. fumigatus* fludioxonil induces a rapid, transient phosphorylation and translocation of the MAP kinase SakA that leads to a tremendous cellular swelling. Fludioxonil blocks growth of germ tubes and hyphae, but it is unable to completely prevent germination of resting conidia. Prolonged incubation in the presence of fludioxonil results in rather odd cellular morphologies. These phenotypic changes are stable as long as the agent is present, but normal growth can be restored after removal of the agent. Apart from their swelling, fludioxonil-treated *A. fumigatus* cells are remarkable because of their large number of nuclei. A block in nuclear division, as recently suggested for fludioxonil-treated *A. nidulans* [17], was not detectable; instead cytokinesis and mitosis seem to be transiently uncoupled, resulting in the accumulation of many more nuclei per cell than normal. These fludioxonil-induced phenotypic changes are dependent on TcsC, since they do not occur in the Δ*tcsC* mutant.

The complete resistance of the Δ*tcsC* mutant to fludioxonil and related fungicides correlates with its high sensitivity to hyperosmotic stress. It has been shown for several plant-pathogenic fungi that fludioxonil mediates its anti-fungal effect by activating the HOG pathway via a Group III HHK [9]. It is therefore conceivable that the characteristic swelling of fludioxonil-treated *A. fumigatus* cells results from a hyperactivation of SakA. This is already detectable after 2 minutes and seems to trigger an uncontrolled increase in the intracellular osmotic pressure. In *A. fumigatus*, TscC is clearly required for the activation of the HOG pathway by both, fludioxonil and hyperosmotic stress. Thus, the inability of the Δ*tcsC* mutant to adapt to hyperosmotic stress and its resistance to fludioxonil both reflect the important role of the TcsC-SakA signaling axis in the control of the internal osmotic pressure of *A. fumigatus*.

The life cycle of *A. fumigatus* is tightly controlled by environmental cues. In contact with air hyphae initiate the formation of conidiophores and the production of conidia. The ‘fluffy’ developmental program impedes sporulation and leads to the massive formation of aerial hyphae and the appearance of white, dome-shaped colonies. Fluffy *A. nidulans* colonies were initially described after treatment with 5-azacytidine [20]. The phenotypical stability of these mutants indicates that a developmental program is permanently activated in these cells. We have recently identified the sesquiterpene alcohol farnesol as a trigger for transient fluffy growth in *A. fumigatus* [21]. In the current study, we observed similar phenotypic switches in response to hypoxia and elevated concentrations of certain divalent cations. The fluffy growth type likely provides an advantage enabling the fungus to survive under certain kinds of stress. The Δ*tcsC* mutant shifts earlier towards this phenotype than the wild type. White, dome-shaped colonies appeared at lower concentrations of farnesol and divalent cations and at less pronounced hypoxia. The earlier adaptation of the mutant does not result in a higher robustness, but seems to be the consequence of a reduced stress resistance. The limited compensatory potential of the fluffy growth was in particular evident at elevated calcium concentrations. The mutant shifts already at 50 mM calcium chloride, but its growth is abolished at 500 mM calcium chloride, when the wild type is still growing well.

**Figure 8:**
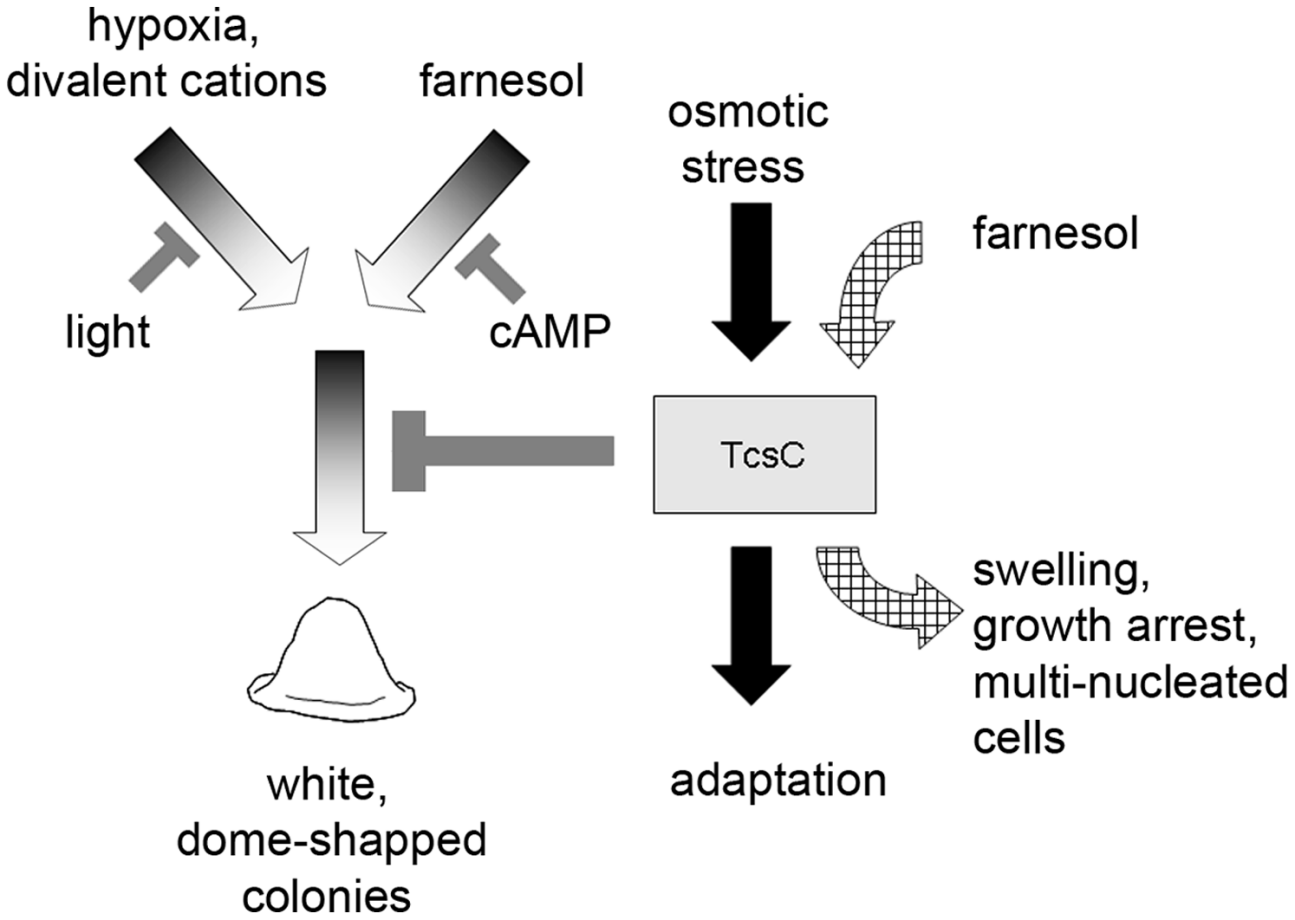
Schematic model of the biological activities of *A. fumigatus* TcsC.

So far, little is known about the mechanisms that underlie the fluffy growth phenotype and cause its peculiar morphological changes. In *A. nidulans* the fluffy growth seems to be controlled by a heterotrimeric G protein that is linked to the cAMP-dependent protein kinase pathway [22], [23]. This and the recent finding that farnesol blocks adenylyl cyclase activity in *Candida albicans* [25] prompted us to study the relevance of the intracellular cAMP level. Addition of cAMP abrogated the farnesol-induced block in sporulation in the wild type, but cAMP was unable to rescue the sporulation defect caused by hypoxia or elevated calcium concentrations. Light is an environmental signal that stimulates sporulation in many fungi. Exposure to light restored normal growth and sporulation under hypoxic conditions and in the presence of elevated concentrations of divalent cations. Thus, light, cAMP and the TcsC protein are factors that impede an activation of the fluffy growth program caused by hypoxia, farnesol or divalent cations (Figure 8).

A stable fluffy *A. fumigatus* mutant secretes more proteases and has an increased angioinvasive growth capacity [26]. This suggests that fluffy hyphae may be well adapted to the specific requirements during infection. In line with this hypothesis, we identified oxygen limitation as another trigger for a fluffy growth. It will be interesting to analyze to what extent the fluffy growth program observed *in vitro* resembles the morphogenetic program that is active during infection.

The Δ*tcsC* mutant shows a normal sensitivity to oxidative, temperature and pH stress as well as clinically relevant anti-fungal agents. On the other hand, TcsC activity is important for the response to a limited array of stress signals including hypoxia (Figure 8). The ability to adapt to oxygen limitation is an essential characteristic of many pathogenic microorganisms. Tco1, the homologous group III HHK in *Cryptococcus neoformans* regulates growth under hypoxic conditions and is also required for virulence [27]. In *A. fumigatus* the situation seems to be different, since we observed no significant attenuation in virulence for the Δ*tcsC* mutant. However, TcsC is required for the anti-fungal activity of fludioxonil and related compounds and may therefore be an attractive target for new therapeutic anti-fungals. Further studies are underway to define the precise mode of action of the TcsC stress sensing pathway and the impact of fludioxonil on growth and survival of *A. fumigatus*.

## Materials and Methods

### Strains Media and Growth Conditions

The *A. fumigatus* strain AfS35, a derivative of strain D141, has been described in [28]. AMM and YG medium were prepared as described [29]. AMM was either supplemented with 1.4 M NaNO_3_ [30] or 0.2 M ammonium tartrate. For hypoxic growth plates were incubated at 37°C in a HERAcell 150i incubator (Thermo Fisher Scientific) adjusted to 5% CO_2_ and the desired oxygen concentration.

### Sequence Analysis and Data Base Searches

Domains were predicted using SMART (http://smart.embl-heidelberg.de/) and alignments were performed using CLUSTAL (http://www.ebi.ac.uk/Tools/msa/clustalw2/).

### Construction of the Δ*tcsC* Mutant Strain

All oligonucleotides used in this study are listed in Table S1. To construct a suitable replacement cassette a 3.5 kb hygromycin resistance cassette was excised from pSK346 using the SfiI-restriction enzyme. The flanking regions of the *tcsC* gene (approx. 900 bp each) were amplified by PCR from chromosomal DNA using the oligonucleotide pairs tcsC-upstream and tcsC-downstream. These oligonucleotides harbor ClaI and Sfi sites. After digestion with ClaI and SfiI, ligation of the three fragments (resistance cassette and flanking regions) yielded a 5.3 kb deletion cassette that was purified using the Wizard SV Gel and PCR Clean-Up System (Promega). The fragment was cloned into the pCR2.1 vector (Invitrogen) using oligonucleotide-derived ClaI sites. A 9.2 kb fragment from the resulting plasmid was linearized with SpeI and used for transformation of *Aspergillus*. The construct used for complementation of Δ*tcsC* was generated by amplifying *tcsC* and its native promoter (1.5 kb region upstream of the gene) from chromosomal DNA using the oligonucleotides tcsC + native promoter-forward and tcsC-reverse. The *gpdA* promoter was excised from the pSK379 vector using EcoRV and NsiI; the latter enzyme generates sticky ends compatible with those generated by PstI. The amplified *tcsC* + native promoter fragment was cloned into this modified version of pSK379 using oligonucleotide-derived NsiI sites. The resulting plasmid was purified as above and used for transformation of the Δ*tcsC* mutant.

*A. fumigatus* protoplasts were generated and fungal transformation was performed essentially as described previously [29]. The resulting protoplasts were transferred to AMM plates containing 1.2 M sorbitol and either 200 µg/ml hygromycin (Roche, Applied Science) or 0.1 µg/ml pyrithiamine (Sigma-Aldrich).

### Genomic DNA Analysis

*A. fumigatus* clones which showed the expected resistance on selective plates were further analyzed by PCR. The correct integration of the deletion cassette was analyzed at the 5′ end using oligonucleotides tcsC-upstream-forward and hph-3-SmaI (PCR1) and at the 3′ end using oligonucleotides trpCt-forward and tcsC-downstream-reverse (PCR2) (Figure S2). To detect the presence of *tcsC* in the complementation mutant, the entire *tcsC* gene was amplified using primers at the 5′ and 3′ ends of the gene (tcsC-forward and tcsC-reverse, PCR3). Primer sequences are listed in Table S1.

### Quantification of Sporulation Efficiency

For each strain tested, three small tissue culture flasks (25 cm^2^; Sarstedt, Nürnbrecht, Germany) with YG agar were inoculated with 4×10^6^ conidia per flask. After incubation for 4 days at 37°C conidia were harvested and counted using a Neubauer chamber.

### Spore Viability Assay

To determine their viability, 2×10^4^ resting conidia were transferred to 1 ml YG medium in a 24 well plate. After overnight incubation at 37°C samples were fixed by addition of 100 µl 37% formaldehyde. The percentage of germinated cells was determined microscopically. These experiments were done in triplicate.

### Protein Extraction and Western Blot

For protein extractions from resting conidia, 75 cm^2^ flasks containing YG agar were inoculated with AfS35 or Δ*tcsC* conidia (in triplicate) and grown at 37°C for 3 days. Conidia were harvested in sterile water and the pellet frozen overnight at –20°C. Frozen conidia pellet was lyophilized overnight at 6°C. The dry pellet was ground with a mortar and pestle in liquid nitrogen. The ground conidia powder was added to 300 µl Laemmlie buffer (2% [w/v] SDS, 5% [v/v] mercaptoethanol, 60 mM Tris/Cl pH 6.8, 10% [v/v] glycerol, 0.02 [w/v] bromophenol blue), heated at 95°C and immediately extracted twice using a Fast Prep 24 (M.P. Biomedical, Irvine, CA) with a speed of 5.5 m/s for 20 s, followed by a final heat denaturation at 95° C for five minutes. 20 µl protein extract was used for SDS-PAGE on 12% SDS gel. Proteins were blotted onto 0.45 µm nitrocellulose membranes and labelled with an α-phospho-p38 MAP kinase antibody (Cell Signaling Technology [#9211], MA, USA). A monoclonal antibody directed against mitochondrial MnSOD (P118-H3) kindly provide by Bettina Bauer was used as a loading control. For protein extractions from germ tubes, 4×10^7^ resting conidia were inoculated in 10 ml AMM and incubated 9 h at 37°C. The germ tubes were treated with 10 µg/ml fludioxonil or 1.2 M sorbitol for 2 min or 20 min, respectively, at 37°C. Protein was then extracted from the cell pellet as above and used for SDS-PAGE and immunoblot in the same manner.

### Phenotypic Plate Assays

Isolated conidia were counted using a Neubauer chamber. For drop dilution assays, a series of tenfold dilutions derived from a starting solution of 1×10^8^ conidia per ml were spotted in aliquots of 1 µl onto plates. These plates were supplemented with the indicated agents and incubated at the indicated temperatures. For quantification of the radial growth, 3 µl containing 3×10^4^ conidia were spotted in the centre of a 9 cm Petri dish. The radius of the colonies was determined over time.

E-test strips of voriconazole, amphotericin B and caspofungin were obtained from Inverness Medical (Cologne, Germany). Each E-test strip was placed onto an AMM agar plate spread with 8×10^5^ conidia. Plates were incubated 36–48 h at 37°C.

Paper disk assays were performed by spreading 8×10^5^ conidia on AMM, Sabouraud, or YG agar plates and placing a sterile paper disk containing fludioxonil, iprodione, or quintozene (Sigma-Aldrich; 46102, 36132 and P8556, respectively), or H_2_O_2_ or tert-butyl hydroperoxide (t-BOOH; Sigma-Aldrich). Plates were incubated 36–48 h at 37°C. Fludioxonil and iprodione were dissolved at 100 mg/ml stock concentrations in DMSO and quintozene was dissolved at 10 mg/ml stock concentration in chloroform. The influence of light was analyzed using an LED light (Osram DOT-it, Osram, Munich, Germany) that was affixed 15 cm above the Petri dish.

### Microscopic Analysis

To visualize the effects of fludioxonil on germ tubes and resting conidia, AfS35 or Δ*tcsC* resting conidia were inoculated in 24-well plates containing 1 ml AMM and glass cover slips. Germ tubes were generated by incubating at 30°C overnight before adding 1 µg/ml fludioxonil. After incubation at 37°C for the indicated times, cells were fixed in 3.7% formaldehyde for five minutes at room temperature. Cover slips were mounted to glass slides in Vecta Shield containing DAPI (Vector Laboratories, Burlingame, California, USA). Cells were then visualized using a Leica SP-5 microscope (Leica Microsystems).

### Infection Experiments

To analyze the impact of TcsC we used an intranasal infection model using immunocompromized female outbred CD-1 mice. Mice were immunosuppressed by intraperitoneal injection of cortisone acetate (25 mg/mouse, Sigma-Aldrich) on days –3 and 0. On day 0 the mice were anesthetized with fentanyl (0.06 mg/kg, Janssen-Cilag, Germany), midazolam (1.2 mg/kg, Roche, Germany) and medetomidin (0.5 mg/kg, Pfizer, Germany) and infected intranasally with 1×10^6^ conidia in 20 µl PBS. Controls received PBS only. Survival was monitored for 14 days. During this period, mice were examined clinically at least twice daily and weighed individually every day. Kaplan-Meier survival curves were compared using the log rank test (SPSS 15.0 software). Mice were cared for in accordance with the principles outlined by the European Convention for the Protection of Vertebrate Animals Used for Experimental and Other Scientific Purposes (European Treaty Series, no. 123; http://conventions.coe.int/Treaty/en/Treaties/Html/123). All animal experiments were in compliance with the German animal protection law and were approved (permit no. 03-001/08) by the responsible Federal State authority and ethics committee.

## Supporting Information

Figure S1 **(A) Schematic drawing of the genomic** ***tcsC*** **gene and the deleted** ***tcsC*** **::** ***hph/tk*** **locus.** Approximately 1 kb of the 5′ and 3′ regions of *tcsC* gene were used for construction of the deletion cassette. The positions of the primers employed for the PCR amplifications and the resulting PCR products (PCR 1-3) are indicated. (B) Equal amounts of genomic DNA of AfS35, Δ*tcsC* and Δ*tcsC*+*tcsC* were used as template for PCR amplification of the regions indicated in panel A (PCR 1-3).
(TIF)

Figure S2 **Resistance of the** Δ***tcsC*** **mutant to iprodione and quintozene.** The sensitivity to iprodione and quintozene was analyzed in drop dilution assays. AfS35 (top) and its Δ*tcsC* mutant (bottom) were spotted on plates without fungicides (panel A) or plates containing either 25 µg/ml quintozene (panel B) or 25 µg/ml iprodione (panel C). Pictures were taken after 48 h at 37°C.
(TIF)

Figure S3 **Impact of fludioxonil during germination of** ***A. fumigatus*** **conidia.** Conidia of *A. fumigatus* strain AfS35 were seeded on glass cover slips and incubated at 37°C in the presence of 1 µg/ml fludioxonil for 28 h (A, B) and 46 h (C to F). After 46 h the medium was replaced by fresh medium. Fungal cells fixed after another 15 h in the absence of fludioxonil are shown in G and H. DAPI stainings are shown in panels A, C, E and G. All bars represent 10 µm.
(TIF)

Table S1 Oligonucleotides used in this study.
(DOC)
