# Supplementary figures and images for: The Two-Component Sensor Kinase TcsC and Its Role in Stress Resistance of the Human-Pathogenic Mold Aspergillus fumigatus
Source: PLoS One. 2012 Jun 4;7(6):e38262. doi: 10.1371/journal.pone.0038262 (PMC3366943; doi:10.1371/journal.pone.0038262)

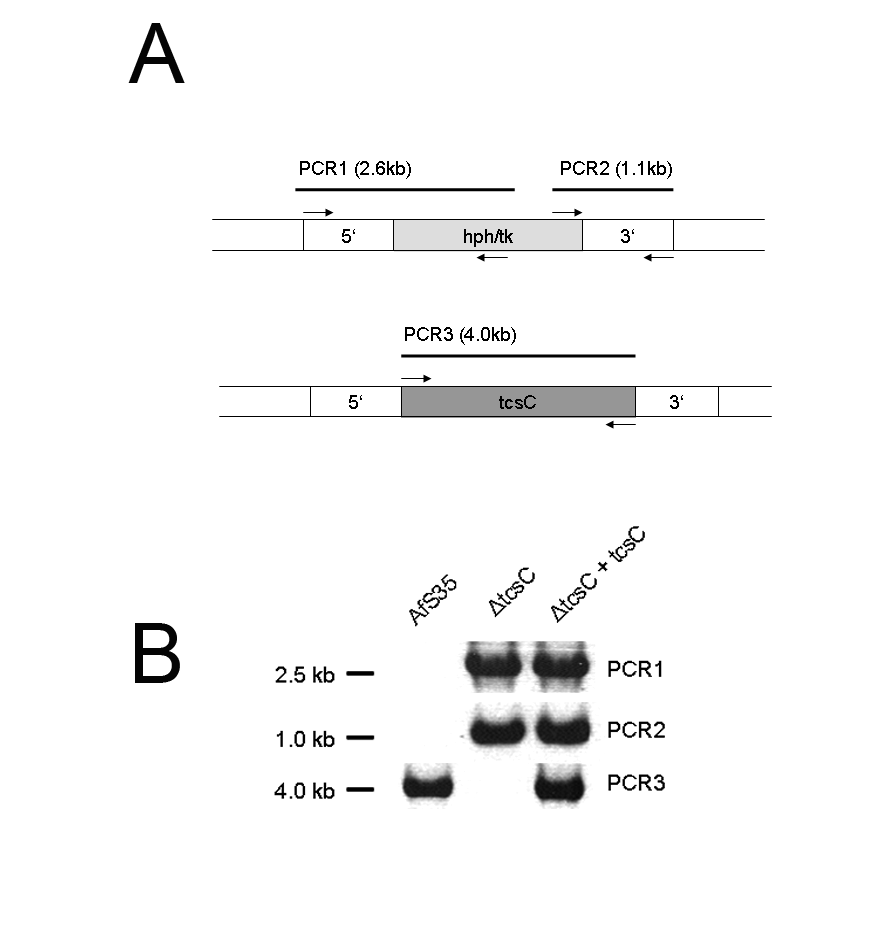

Supplement: Figure S1 — (A) Schematic drawing of the genomic tcsC gene and the deleted tcsC :: hph/tk locus. Approximately 1 kb of the 5′ and 3′ regions of tcsC gene were used for construction of the deletion cassette. The positions of the primers employed for the PCR amplifications and the resulting PCR products (PCR 1-3) are indicated. (B) Equal amounts of genomic DNA of AfS35, ΔtcsC and ΔtcsC+tcsC were used as template for PCR amplification of the regions indicated in panel A (PCR 1-3). (TIF) [file pone.0038262.s001.tif]

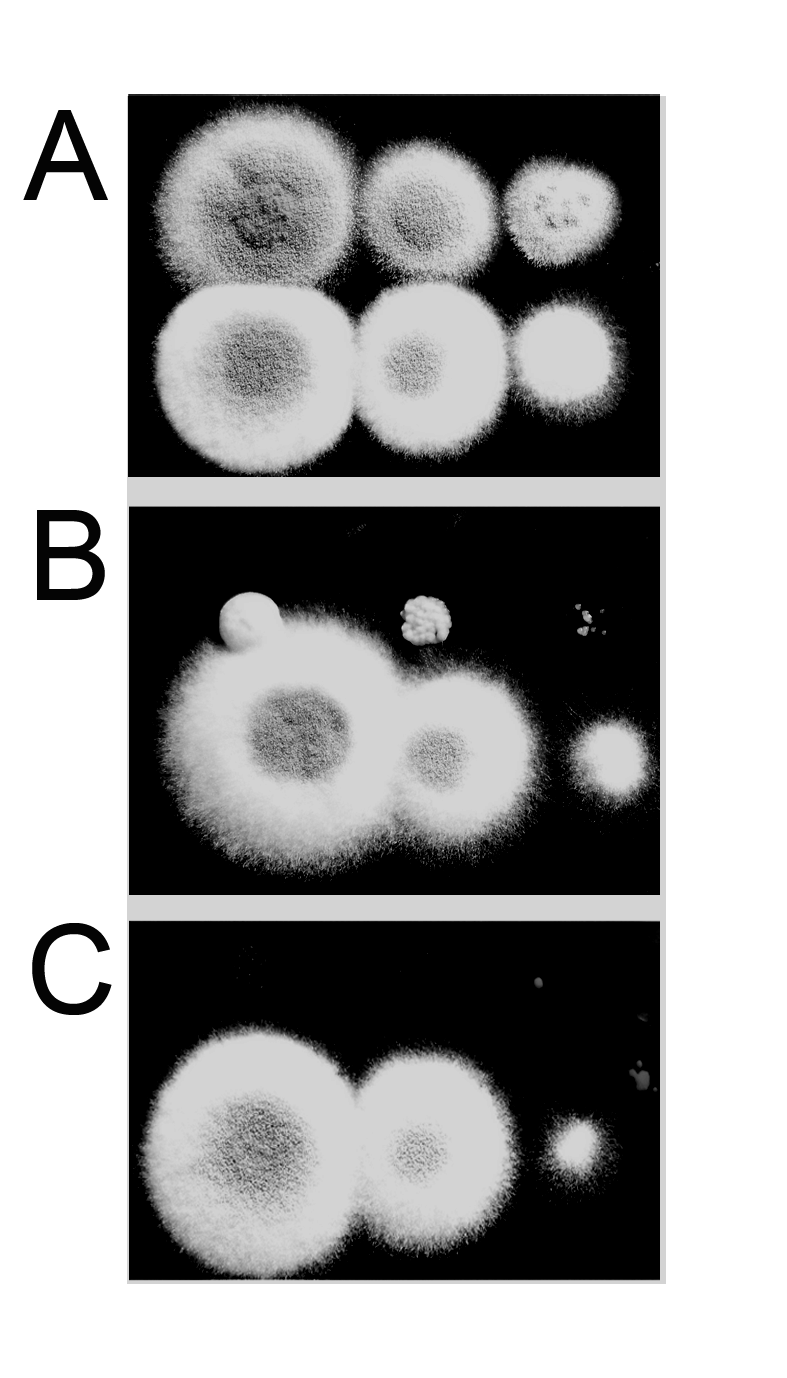

Supplement: Figure S2 — Resistance of the ΔtcsC mutant to iprodione and quintozene. The sensitivity to iprodione and quintozene was analyzed in drop dilution assays. AfS35 (top) and its ΔtcsC mutant (bottom) were spotted on plates without fungicides (panel A) or plates containing either 25 µg/ml quintozene (panel B) or 25 µg/ml iprodione (panel C). Pictures were taken after 48 h at 37°C. (TIF) [file pone.0038262.s002.tif]

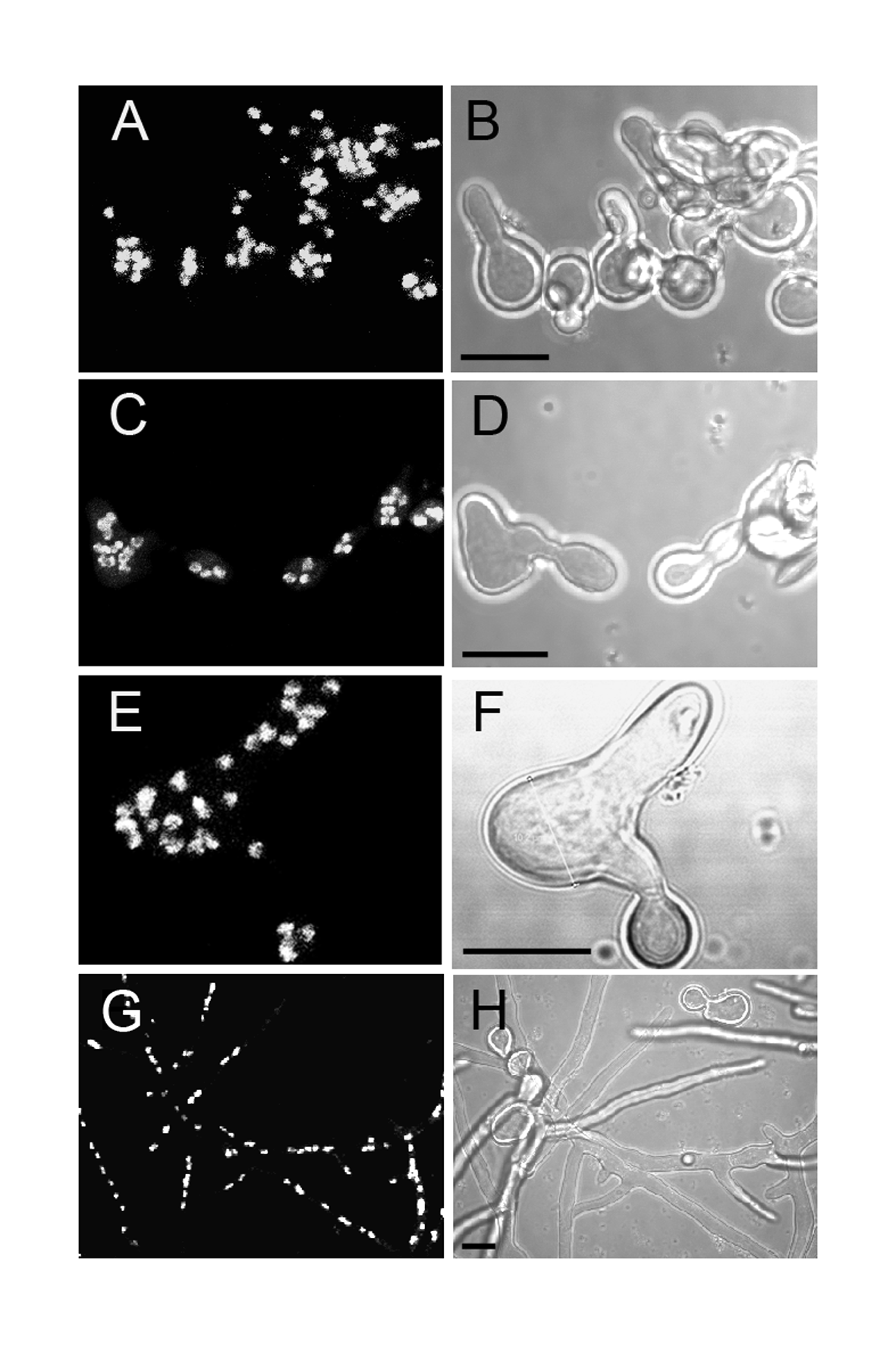

Supplement: Figure S3 — Impact of fludioxonil during germination of A. fumigatus conidia. Conidia of A. fumigatus strain AfS35 were seeded on glass cover slips and incubated at 37°C in the presence of 1 µg/ml fludioxonil for 28 h (A, B) and 46 h (C to F). After 46 h the medium was replaced by fresh medium. Fungal cells fixed after another 15 h in the absence of fludioxonil are shown in G and H. DAPI stainings are shown in panels A, C, E and G. All bars represent 10 µm. (TIF) [file pone.0038262.s003.tif]
