# Supplementary material for: The Two-Component Sensor Kinase TcsC and Its Role in Stress Resistance of the Human-Pathogenic Mold Aspergillus fumigatus
Source: PLoS One. 2012 Jun 4;7(6):e38262. doi: 10.1371/journal.pone.0038262 (PMC3366943; doi:10.1371/journal.pone.0038262)
Supplement: Table S1 — Oligonucleotides used in this study. (DOC) [file pone.0038262.s004.doc]

**Table S1:** Oligonucleotides used in this study.

|  | **Primer Name** | **Sequence** | **Figure S1** |
| --- | --- | --- | --- |
| **To create Δ*tcsC* knockout mutant** | tcsC-upstream-forward | cca act cac aac tca atc gc | PCR1 forward |
|  | tcsC-upstream-reverse | aaa tcg atg gcc tga gtg gcc tgt gtc tgt tgt aaa tta gt |  |
|  | tcsC-downstream-forward | aaa tcg atg gcc atc tag gcc gaa gac aat cgc tta ttg ga |  |
|  | tcsC-downstream-reverse | acg cgc ggt agg gta caa aa | PCR2 reverse |
| **To create the complemented strain** | tcsC-forward | aca atg act ggc gca gac gag acg | PCR3 forward |
|  | tcsC + native promoter-forward | ctg cag tat cgt cgg tat tat tca |  |
|  | tcsC-reverse | ttc tca tac ggc ctt tgg aga gcg | PCR3 reverse |
| **Within hph cassette** | trpCt-forward | cag aat gca cag gta cac ttg | PCR2 forward |
|  | hph-3-reverse | tgg cta aga tcg gcc gca | PCR1 reverse |
